# Subtle changes in plant diversity in the Bavarian Alps over the past eight decades

**DOI:** 10.1002/ece3.70035

**Published:** 2024-09-02

**Authors:** Meredith A. Zettlemoyer, Svenja Munck, Peter Poschlod, Sergey Rosbakh

**Affiliations:** ^1^ Division of Biological Sciences University of Montana Missoula Montana USA; ^2^ Department of Ecology and Conservation Biology Universitat Regensburg Regensburg Germany; ^3^ Department of Plant and Environmental Sciences University of Copenhagen Copenhagen Denmark

**Keywords:** (sub)alpine vegetation, Alps, community composition, functional diversity, global change, grassland, historical record, re‐survey, richness, stability

## Abstract

Historical resurveys represent a unique opportunity to analyze vegetation dynamics over longer timescales than is typically achievable. Leveraging the oldest historical dataset of vegetation change in the Bavarian Alps, Germany, we address how environmental conditions, vegetation composition, and functional diversity in the calcareous grasslands of the Schachen region have changed across different elevational ranges over an 83‐year timeframe. We document changes in regional average temperature and precipitation. We use indicator values (IV) for species' ecological preferences and their palatability to grazers to infer local conditions (temperature, soil moisture/fertility, and grazing regime). We further estimate changes in temporal beta‐diversity and functional trait community composition between historical (1936) and contemporary (2019) surveys in two elevational (subalpine and alpine) belts. Both subalpine and alpine sites became drier; subalpine sites also became warmer with more palatable plants. Species occurrence and abundance in the Schachen region has not changed substantially over time despite changing macroclimate and local environmental conditions under anthropogenic change. Yet these grasslands have experienced several “invisible” changes in functional composition over the past 80 years. As the Schachen has become drier, species with traits related to drought tolerance and animal‐based dispersal have increased in dominance. Specifically, in alpine sites, community‐weighted means revealed that with low fecundity, higher potential for endo‐ and epizoochory (seed dispersal via animal gut and fur, respectively), higher foliar frost tolerance, and deeper dormancy increased in dominance. Similar trends were found for increasing dominance of low fecundity, epizoochorous species in subalpine sites. Vegetation data from resurveying historical plots in combination with changes in local conditions, classic biodiversity indices, and functional trait indices can provide more holistic insights into changes in the environment and potential impacts of those environmental changes on long‐term plant community and functional diversity.

## Graphical abstract

We leverage the oldest vegetation resurvey in the Bavarian Alps to address how environmental conditions, vegetation composition, and functional diversity have shifted in the calcareous (sub)alpine grasslands of the Schachen region between 1936 and 2019. The Schachen is becoming drier across both subalpine and alpine grasslands; subalpine sites are also becoming warmer and more palatable to grazers as grazing pressure has decreased. Although species composition has not changed substantially in the Schachen over the past 83 years, these grasslands have experienced subtle changes in functional diversity as species with traits related to drought tolerance and animal‐based dispersal have increased in dominance. This study represents a unique use of historical resurveys to measure temporal changes in vegetation abundance and functional structure that may not be reflected in measurements of change in species occurrence.

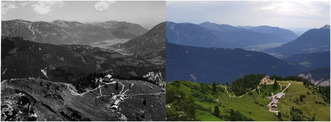

## 1 INTRODUCTION

Mountain landscapes, consisting of a mosaic of pastures, meadows, forests, and diverse microhabitats that span montane to alpine vegetation belts (Körner & Hiltbrunner, 2021), serve as a reservoir of biodiversity (Britton et al., 2009). They harbor specialized biota, including species adapted to cold, harsh environments at higher elevations; species tolerant of intense competition in more densely vegetated, lower‐elevation habitats; and endemic species resulting from geographic isolation and glacial refugia (Körner, 1995; Razgour et al., 2015). The topographical complexity of mountainous regions (e.g., spatial variability in slope and aspect) also results in steep spatial gradients in environmental conditions (Graae et al., 2018), allowing many species to coexist in a small area and promoting high spatial turnover of spatial species composition (Bjorkman et al., 2018; Elmendorf et al., 2012; Jurasinski & Kreyling, 2007). Mountain landscapes may also be subject to high temporal turnover in species composition because they experience anthropogenic changes, including climate change, land use change, grazing pressure, and eutrophication (higher soil nutrient loads due to a combination of nitrogen deposition and fertilization) (Alexander et al., 2016; Dainese et al., 2017; Petitpierre et al., 2016).

Mountain habitats are particularly vulnerable to anthropogenic changes. They often experience more dramatic warming than lower elevation habitats (Rumpf et al., 2022). For example, in the European Alps, temperatures have increased by roughly twice the global mean (Kotlarski et al., 2023). Temperate mountains have also been subject to changes in the management of herbivores and increased development and recreation (Körner, 1999). Historically, land scarcity caused humans to take livestock to graze in grasslands above treeline in temperate mountain ranges such as the European Alps (Gilck & Poschlod, 2019; Mayer & Erschbamer, 2017), the Scottish Highlands (Van der Wal et al., 2003), and the Spanish Pyrennes (Muñoz‐Ulecia et al., 2024). This shift in grazing management increases herbivore pressure and nitrogen deposition impacts in alpine meadows (Van der Wal et al., 2003), although alpine grazing pressure has since declined with the onset of modern dairy farming (Marini et al., 2011). Climate and land use change can result in range expansions of more competitive, shrubby, thermophilous, and/or non‐native species into higher‐elevation habitats, particularly if disturbances like grazing open new niches (Gottfried et al., 2012; Iseli et al., 2023; Lamprecht et al., 2018; Rosbakh et al., 2014; Steinbauer et al., 2018). Climate and land use change can also result in local extinction of alpine species (Guisan & Theurillat, 2000; Pauli et al., 2012; Steinbauer et al., 2018) and homogenization of alpine regions (Haider et al., 2018; Jurasinski & Kreyling, 2007). However, the effects of climate and land use change on vegetation composition can vary with elevation (Saatkamp et al., 2023). For this example, we refer to three elevational belts: subalpine habitat (forest and grasslands located below treeline), the low‐elevation alpine meadows (grasslands located just above treeline), and higher‐elevation alpine habitats (harsher, colder habitats located above treeline at a greater distance from subalpine species pools). In this case, low‐elevation alpine meadows might experience increases in plant cover and diversity due to subalpine species readily migrating into suitable habitat just above treeline. Low‐elevation alpine meadows might also show increasing abundance of already‐present thermophilic species. In contrast, high‐elevation alpine meadows might experience less colonization by subalpine species than lower‐elevation alpine meadows due to dispersal limitation, longer distances from the subalpine species pools, and more unfavorable conditions for species establishment (Dirnböck et al., 2011; Rosbakh et al., 2014; Smithers et al., 2020). Alternatively, low‐elevation alpine meadows could experience less diversity change if there are few available gaps for newly arrived species (Vittoz et al., 2009). However, few studies have addressed changes in vegetation across multiple elevational belts within mountain habitats (Rosbakh et al., 2014).

Monitoring changes in montane vegetation requires long‐term monitoring and knowledge of baseline conditions (Kapfer et al., 2016). However, we often lack the comprehensive and spatiotemporally replicated datasets that are required to study biodiversity change through time (Lindholm et al., 2021). Historical biodiversity records (e.g., recordings from permanent plots or phytosociological surveys) can provide such an indicator of baseline conditions prior to intensive anthropogenic change because they document the presence of alpine species that are at high risk of loss under current and future conditions. However, very few long‐term biodiversity monitoring programs exist in montane environments, and those that do are often restricted to the last few decades (e.g., GLORIA, which began operations in the early 2000s; www.gloria.ac.at). Alternatively, we can revisit plots set up by independent parties for one‐time botanical surveys in the past (historical plots; Kapfer et al., 2016). These resurveys have gained attention in recent years (Hédl et al., 2017) despite their limitations (e.g., these datasets are often only based on two or three timepoints and are often missing metadata; Tessarolo et al., 2017). Here, we highlight three benefits of historical resurveys for quantifying biodiversity change over time; this list is not exhaustive. First, their potentially broad temporal scales can allow us to test for temporal variation in species composition, or changes in species occupancy over time. In particular, the Temporal Beta‐Diversity Index (TBI; Legendre, 2019) can decompose changes in species composition into losses and gains at particular sites and potentially guide management by pointing out locations declining in diversity vs. those that are temporally stable. Second, resurveys of historical plots along elevational gradients can detect altered dynamics in different vegetation belts (e.g., subalpine vs. alpine; Gazol et al., 2017; Kapfer et al., 2016; Løkken et al., 2020; Spasojevic & Suding, 2012; Virtanen et al., 2010; Wilson & Nilsson, 2009). Finally, historical resurveys can be used to infer changes in community functional composition (e.g., shifts in functional trait mean and variation values).

Shifts in functional composition should reflect the main adaptive strategies in response to changing environmental conditions (Cerabolini et al., 2010; de Bello et al., 2013; Magurran, 2021; McGill et al., 2006). For example, increased grazing might result in decreasing leaf nitrogen content and thinner leaves (i.e., less palatable species; Freitag et al., 2020). In mountains, we might expect alpine species adapted to short growing seasons with frequent and severe freezing events, and shallow, nutrient‐poor soils to disappear from the community as conditions warm, seasons lengthen, and nutrient inputs increase. These factors might correspond with the disappearance of short species with smaller leaves and lower specific leaf area (SLA), as many alpine plants grow low to the ground to cope with low temperatures and have thicker leaves to minimize water loss and frost damage (Cruz‐Maldonado et al., 2021; Onoda et al., 2017; Wright et al., 2004).

Here, we ask how vegetation composition and functional diversity changes over an 83‐year period (1936–2019) across two elevational belts in the Wetterstein Mountains (North Calcaerous Alps), Germany, using resurveys of semi‐permanent plots. Baseline data were recorded in 1936–1937 by Niilo Söyrinki (Söyrinki, 1954) as a series of phytosociological surveys (‘releves’), and a resurvey was conducted in 2019. We aim to address how environmental conditions (temperature, soil moisture and fertility, and grazing regime), species richness and community composition, and community functional trait composition have changed across subalpine and alpine communities over the 83‐year period. We tested three hypotheses:

Both subalpine and alpine plots become warmer, drier, and more nutrient‐rich with more palatable (i.e., increasing leaf N content and thinner leaves) plants over time due to climate warming, increased nitrogen eutrophication, and declines in traditional grazing at high elevations in the study area.

Temporal beta‐diversity in both subalpine and alpine site experience species losses and gains over time as a result of the putative environmental changes suggested in H1. Specifically, we hypothesize that, in accordance with expected increased warming and eutrophication and decreased grazing pressure in the Schachen, species losses will drive any observed changes in temporal beta‐diversity.

Community‐level functional trait composition shifts to favor traits associated with “thermophilisation” (i.e., warmer and drier conditions; e.g., increased plant height, increased specific leaf area (thinner leaves), and decreased frost‐resistance), land use changes (e.g., increased specific leaf area and decreased endo‐ and epizoochory in response to decreased grazing pressure), and increased eutrophication (e.g., increased specific leaf area and leaf nitrogen content; Table 1).

**TABLE 1:** Plant functional traits (grouped as vegetative or generative), units, their function (following de Bello et al., 2013; Weiher et al., 2009), and their expected response to changes in temperature, eutrophication, land use (largely increased grazing by cattle and sheep).

Trait (units)	Function	Response
*Vegetative*		
Plant height (m)	Competitive ability, stress tolerance, stress avoidance	Temperature (+), eutrophication (+), land use change (+/−)
Specific leaf area (SLA) (m^2^ kg^−1^)	Relative growth rate, resource acquisition, stress tolerance, leaf longevity	Temperature (+), eutrophication (+), land use change (+)
Leaf frost tolerance (%)	Leaf longevity, stress tolerance	Temperature (+/−)
Leaf nitrogen (N) content (mg g^−1^)	Maximum photosynthetic rate, fecundity	Eutrophication (+)
*Generative*		
Seed mass (mg)	Dispersal distance, longevity in seed bank, establishment success	Temperature (+), eutrophication (+), land use change (+/−)
Seed production (numeric)	Establishment success, fecundity	Temperature (+/−), eutrophication (+), land use change (+/−)
Seed terminal velocity (falling speed) (m s^−1^)	Dispersal distance	Temperature (+/−), eutrophication (+/−), land use change (+/−)
Seed dormancy (0/1)	Establishment success, longevity in seed bank	Temperature (+)
Seed dispersal via attachment to fur (epizoochory) or animal ingestion (endozoochory)	Dispersal distance	Land use change (−)

*Note*: + indicates expected positive response; − indicates expected negative response; +/− indicates an expected response but no a priori hypothesis for direction.

## 2 MATERIALS AND METHODS

### 2.1 Study site

The Schachen area of the Wetterstein Mountains, Bavarian Alps, Germany (47.4203727, 11.1135369) extends from 1150 to 2630 m a.s.l., with treeline located at approximately 1800–1850 m. The Schachen area experiences a typical mountain climate, showing a large decrease in mean annual air temperature from 7.2°C in Garmisch‐Partenkirchen (708 m a.s.l.) to −4.3°C at the Zugspitze, a 2962 m high summit 10.5 km west of the Schachen area. Mean annual precipitation in this area ranges between 1100 and 2150 mm year^−1^ (Deutscher Wetterdienst, www.dwd.de).

The non‐forest vegetation mainly consists of species‐rich calcareous grasslands on nutrient‐poor, shallow soils. The grasslands of the montane belt (800–1400 m) are dominated by tall forbs and grasses, which are replaced by sedges, short‐statured herbs, and dwarf shrubs in the subalpine (1400–1900 m) and alpine (1900–2600 m) belts as elevation increases.

**FIGURE 1:**
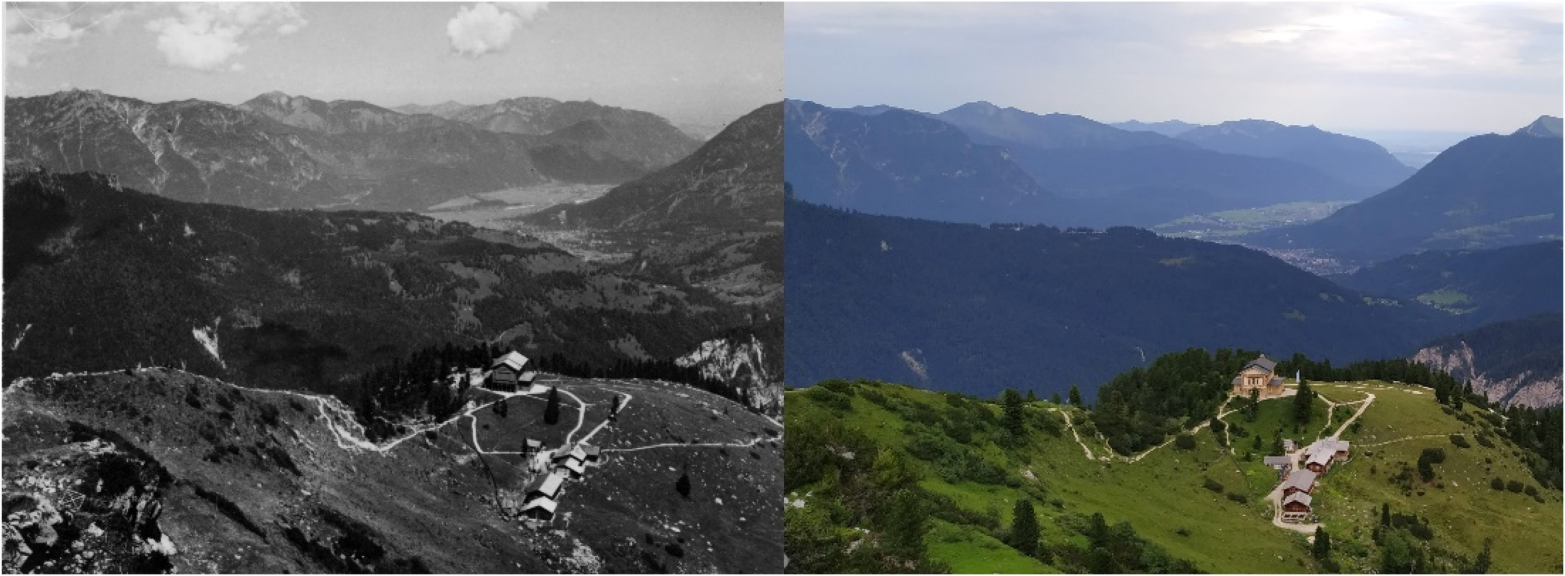
The Schachenhaus (1866 m a.s.l.) in 1936 (left) and 2019 (right).

The Schachen's subalpine and alpine grasslands are grazed by cattle (mainly montane‐subalpine belts, May–September) and sheep (almost exclusively alpine belt, mid‐July to late‐September), beginning in 1726. The density of grazing in the subalpine grasslands has decreased over time (100 animals total in 1726, 80 in the 1930s, 50 in the 2010s; pers. comm., Garmisch‐Partenkirchen district administration). Two residences, the Meilerhütte (2366 m a.s.l.) and the Schachenhaus (1867 m a.s.l.), were constructed in the late 1800s, and the area has since opened to recreation (Figure 1).

### 2.2 Historical dataset

Niilo Söyrinki, a Finnish botanist (1907–1991), surveyed 88 plots across the Schachen in 1936–1937 (Söyrinki, 1954), following the Hult‐Sernander‐Du Rietz scale (Du Rietz, 1921). In 2019, we resurveyed 43 phytosociological plots spanning seven vegetation communities defined by Söyrinki, following methods in Braun‐Blanquet (1966) (see Table S1 for variable conversion between survey methods). The seven communities can be broadly categorized as subalpine (1800–1950 m a.s.l.) or alpine (2200–2340 m a.s.l.) (Table S2; Figure S1). Sample sizes did not allow for analyses of trends in species or trait composition within each of the seven community types. Historical surveys included precise descriptions of landmarks, elevation, and topography, allowing reliable re‐identification of “semi‐permanent” plots in the field (Lawesson, 2000; Rosbakh et al., 2014). This approach, without precise georeferencing data, is commonly used for historical relevés (Bakker et al., 1996; Hédl, 2004; Kudernatsch, 2005; Ross et al., 2010; Windmaiβer & Reisch, 2013). Although the situation of the plots may not be precise, these semi‐permanent plots were in the same locality and community as the original plots (Rosbakh et al., 2014). Plot sizes ranged from 1 to 25 m^2^; we retained historical plot size during re‐surveys. We excluded historical plots in areas that are frequently disturbed by descending rocks, floods, and avalanches. Plant taxonomy was unified using nomenclature in Schönfelder and Bresinky (1990).

### 2.3 Environmental data

We obtained data on air temperature (2 m above the ground) and precipitation from the closest weather station ‘Zugspitze’ located 11 km west of the Schachen area at 2962 m a.s.l. (www.dwd.de). We averaged temperature and precipitation data across the growing season, which we considered the growing season to be April–September, during which the majority of (sub)alpine plants grow, flower, fruit, and senesce.

### 2.4 Plant functional trait data

We selected 10 functional traits for each of the 194 species recorded in both the historical (1936) and recent (2019) surveys. Each trait potentially affects plant fitness under environmental changes in upland ecosystems (Table 1; Pellissier et al., 2010; Rosbakh et al., 2022). The traits were sampled either in the study communities or in plots with similar ecological conditions (e.g., elevation, soil properties, grazing). For trait measurements, we collected 10–30 individual plants per species (depending on the trait of interest), collected at least 2 m away from each other, from the site where the species is most abundant (i.e., under optimal ecological conditions; Rosbakh et al., 2022). This approach does not account for intraspecific trait variability, but instead quantifies a fixed species mean trait value. At this small spatial scale, we do not anticipate substantial amounts of intraspecific trait variability. Trait measurements followed standardized protocols (Kleyer et al., 2008; Pérez‐Harguindeguy et al., 2013). Seed dormancy data were obtained from Baskin and Baskin (2014), Rosbakh et al. (2020), and Nikolaeva et al. (1985).

### 2.5 Statistical analysis

#### 2.5.1 Environmental change over eight decades

To assess environmental change at the study site, we first fitted two separate ordinary least squares regressions in the *nlme* R package (Pinhiero et al., 2023) to test for temporal trends in mean growing season temperature and precipitation. We included both linear and quadratic fixed effects of year in the models to account for potential nonlinearity in the climate data. We note that this approach does not account for potential differences in climate change between subalpine vs. alpine sites, but finer‐scale climate data (at the plot level) is not currently available. Instead, we use ecological indicator values (see below) to assess changes in local conditions of subalpine vs. alpine sites based on shifts in local species' preferences.

We estimated changes in local soil conditions for subalpine vs. alpine sites during the study period using Landolt Indicator Values (IV). Landolt's IVs aim to semi‐quantitatively describe the most frequent association of a given set of species with environmental conditions (Scherrer & Körner, 2011) Landolt's IVs score the strength of a species' relationship with local environmental variables on a scale of 1 (low) to 5 (high). These scores are then weighted by each species' niche breadth, allowing for a community mean estimate of Landolt's IVs (Ivanova & Zolotova, 2023). We used Landolt's T as a proxy for mean soil and surface temperature after snowmelt, where higher values indicate warmer growing season conditions (Landolt et al., 2010; Scherrer & Körner, 2011). Landolt's F is a proxy for soil moisture, with higher values indicating wetter growing season conditions. Landolt's N is a proxy for soil fertility, with higher values indicating high soil nutrient levels during the growing season. IVs are strongly correlated with directly measured soil temperature (Scherrer & Körner, 2011), nutrients (N, P, and K) and moisture (Rosbakh & Poschlod, 2021) in montane ecosystems. We opted for these proxies due to the lack of corresponding long‐term in situ observations during the study period.

We used an indicator value of forage quality (FQ; Briemle et al., 2002), to infer potential long‐term changes in grazing regime at subalpine vs. alpine sites. FQ is a rating of plant palatability based on field observations ranging from 1 (low forage quality) to 9 (high forage quality). FQ has been shown to be a reliable predictor for cattle (Pauler et al., 2020) and sheep plant species selection (Mládek et al., 2013). We hypothesized that changes in grazing density and/or intensity would result in corresponding changes in FQ values over time (e.g., smaller FQ values at more intensive grazing). Values for several (sub)alpine species missing in Briemle et al. (2002) were supplemented using information about their forage quality in Stebler and Schröter (1889).

For every pair of historical‐recent vegetation plots we calculated the community‐weighted mean IV using the *fd* R package (Laliberté et al., 2014). Changes in IV‐based environmental conditions in the subalpine and alpine plots over the past eight decades were estimated using linear mixed models. We conducted separate models for each environmental factor (temperature, soil moisture, nutrients, and forage quality). Each model included the relevant IV as the response variable and survey year (1936 vs. 2019) as the predictor. We included affiliation with Söyrinki's seven vegetation communities as a random factor. Because of the relatively small number of replicate plots and distinct ecological conditions (e.g., environmental factors, size and composition of species pools), all analyses were conducted for subalpine and alpine sites separately. All model assumptions were met in all cases.

#### 2.5.2 Temporal changes in beta‐diversity

Temporal changes in species occurrence and abundance in subalpine vs. alpine sites were estimated using the Temporal Beta‐Diversity Index (TBI; Legendre, 2019; Lindholm et al., 2021). TBI is a dissimilarity index (Sørensen for presence/absence and Ružička for abundance) that measures changes in community composition between two points of time and decomposes dissimilarities (turnover) into loss and gain components. TBI also tests for significant differences between gains and losses, thereby indicating the overall direction of change in assemblages. We calculated TBI using the *TBI* and *tpaired.krandes*t functions from *adespatial* R package (Dray et al., 2019) under 9999 permutations.

#### 2.5.3 Temporal changes in community functional trait composition

To assess potential changes in functional composition of subalpine vs. alpine sites between 1936 and 2019, we calculated community‐weighted means (CWMs) and functional diversity (FD), two commonly used metrics to infer community assembly rules (Ricotta & Moretti, 2011). The CWM of a community is the average trait value weighted by species relative abundance. It mainly reflects the trait value of the dominant species in a community and thus describes the main adaptation strategy to local environmental conditions (de Bello et al., 2021). FD reflects trait convergence or divergence (i.e., a decrease or increase in trait diversity compared to a null expectation) and was calculated using Rao's quadratic entropy (Rao, 1982). For the CWM and FD calculations, we used a species abundance table from both historical and recent surveys, a functional trait table consisting of the 10 traits for all 187 species found in the 1930s and 2010s, and an environmental variables table consisting of survey year and vegetation type (subalpine vs. alpine). The changes in CWM and FD values between the historic and recent surveys were estimated using linear mixed models as described for IVs above.

All statistical analyses were conducted in R 4.3.0 (R Core Development Team, 2023).

## 3 RESULTS

### 3.1 Environmental change in the Schachen

**FIGURE 2:**
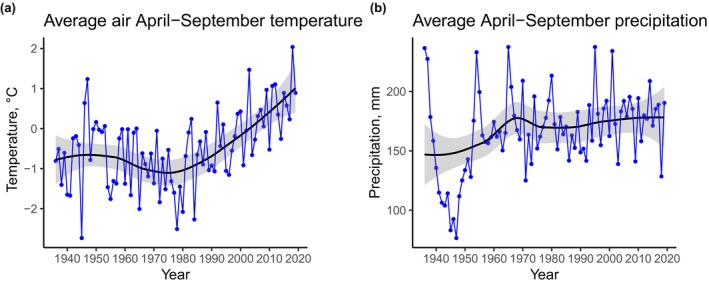
Growing season temperature increases while precipitation remains stable over eight decades in the Schachen area. Changes in average growing season (April–September) (a) air temperature (°C) and (b) precipitation (mm year^−1^). Blue points show annual means, blue lines show interannual variability, the black regression line shows climatic trends from 1936 to 2019, and shaded areas show 95% CIs.

April–September temperatures increased by approximately 2°C over the past eight decades, with a distinctive linear increase from the 1980s onwards (Table S3; Figure 2a). April–September precipitation displayed relatively high intradecadal variability but did not change significantly during the study period (Table S3; Figure 2b).

**FIGURE 3:**
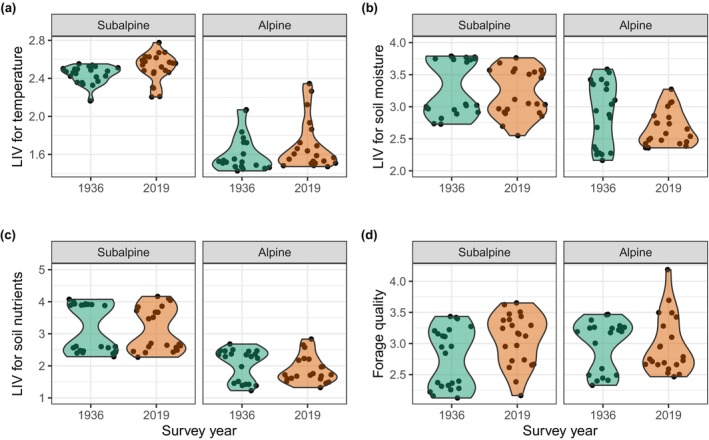
Subalpine plots become warmer, drier, and more palatable over time. Changes in Landolt Indicator Values (LIVs) between 1936 and 2019 for (a) soil and above‐ground temperature (Landolt's T), (b) soil moisture (Landolt's F), (c) soil nutrients (Landolt's N), and (d) plant forage quality (Briemle et al.'s FQ value) in subalpine vs. alpine plots. Asterisks indicate statistically significant (*p* < .05) differences between the historic (1936) and recent (2019) surveys; n. s. – not significant.

Changes in Landolt Indicator Values (IVs) revealed moderate but significant thermophilisation of subalpine plots (Table S4). Landolt's T increased from 2.4 ± 0.1 in 1936 to 3.1 ± 0.1 in 2019 (Figure 3a). In contrast, local thermal conditions did not change significantly in the alpine plots. Both subalpine and alpine plots demonstrated weak yet significant decreases in soil moisture, as shown by a decrease in Landolt's F from 3.1 ± 0.2 to 3.0 ± 0.1 and from 2.9 ± 0.2 to 2.7 ± 0.1 from 1936 to 2019 in subalpine vs. alpine plots, respectively (Figure 3b). Soil fertility (Landolt's N) did not change significantly during the study period (Figure 3c). Subalpine, but not alpine, plots increase in forage quality over time, with an increase in FQ from 2.9 to 3.2 between 1936 and 2019 (Figure 3d).

### 3.2 Temporal changes in beta‐diversity

**FIGURE 4:**
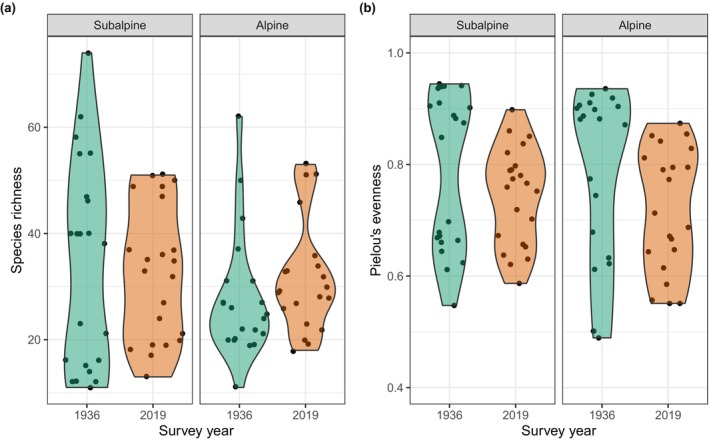
Temporal changes in (a) species occurrence and (b) abundance as estimated with the temporal beta‐diversity index (Legendre, 2019). Asterisks indicate statistically significant (*p* < .05) differences over time between the historic and recent plots; n. s. – not significant.

We detected no temporal turnover in species occurrence in either subalpine (losses = 0.23, gains = 0.26, *p* = .50) nor alpine plots (losses = 0.21, gains = 0.29, *p* = .07; Figure 4a). Species abundance decreased in subalpine plots (losses = 0.42, gains = 0.29, *p* = .003; Figure 4b), while species abundance remained unchanged in alpine plots (losses = 0.34, gains = 0.39, *p* = .11; Figure 4b).

### 3.3 Temporal changes in community functional trait composition

Comparisons of community‐weighted means (CWMs) and Rao's functional diversity (FD) between the historic and the recent surveys revealed several changes in functional trait composition in both subalpine and alpine sites (Table 2). Using CWMs, the dominance of species producing fewer seeds with a high potential for epi‐ and endozoochory significantly moderately increased in alpine sites over the last eight decades. Similar trend for seed production and epizoochory was detected for the subalpine sites. Additionally, species with higher values for foliar frost‐tolerance and dormancy depth (i.e., species better adapted to harsh, cold environments) increased their dominance in alpine sites.

**TABLE 2:** Changes in community functional trait composition as deduced by changes in community‐weighted means and Rao's functional diversity.

Trait	Community‐weighted mean				Functional diversity			
	Subalpine		Alpine		Subalpine		Alpine	
	1936	2019	1936	2019	1936	2019	1936	2019
Plant height	0.21 ± 0.4 a	0.23 ± 0.4 a	0.07 ± 0.02 a	0.07 ± 0.02 a	0.67 ± 0.18 a	0.78 ± 0.18 a	0.10 ± 0.05 a	0.18 ± 0.05 a
Specific leaf area	20.5 ± 0.5 a	21.1 ± 0.5 a	17.1 ± 2.1 a	15.9 ± 2.1 a	1.06 ± 0.28 a	1.04 ± 0.28 a	**0.69 ± 0.08 a**	**0.85 ± 0.08 b**
Leaf N content	2.4 ± 0.1 a	2.4 ± 0.1 a	2.2 ± 0.2 a	2.1 ± 0.2 a	1.08 ± 0.15 a	1.15 ± 0.15 a	0.89 ± 0.15 a	0.92 ± 0.15 a
Leaf frost resistance	55.7 ± 3.2 a	56.6 ± 3.2 a	**41.1 ± 8.1 a**	**33.9 ± 8.1 b**	0.99 ± 0.08 a	0.94 ± 0.08 a	**0.77 ± 0.1 a**	**1.01 ± 0.1 b**
Seed mass	1.2 ± 0.2 a	1.1 ± 0.2 a	0.58 ± 0.1 a	0.57 ± 0.1 a	0.92 ± 0.13 a	0.93 ± 0.13 a	0.58 ± 0.06 a	0.57 ± 0.06 a
Seed production (log (*x* + 0.1)‐transformed)	**6.4 ± 0.4 a**	**5.9 ± 0.4 b**	**5.2 ± 0.3 a**	**4.9 ± 0.3 b**	**1.10 ± 0.46 a**	**0.59 ± 0.46 b**	**0.04 ± 0.01 a**	**0.02 ± 0.01 b**
Seed terminal velocity	2.1 ± 0.2 a	2.1 ± 0.2 a	1.7 ± 0.1 a	1.7 ± 0.1 a	0.94 ± 0.11 a	0.86 ± 0.11 a	0.69 ± 0.19 a	0.70 ± 0.19 a
Potential for epizoochory	**68 ± 7 a**	**73 ± 7 a**	**77 ± 1 a**	**88 ± 1 b**	1.15 ± 0.34 a	1.05 ± 0.34 a	0.52 ± 0.04 a	0.59 ± 0.04 a
Potential for endozoochory	**0.4 ± 0.1 a**	**0.5 ± 0.1 b**	0.3 ± 0.1 a	0.3 ± 0.1 a	0.76 ± 0.18 a	0.76 ± 0.18 a	0.85 ± 0.15 a	0.85 ± 0.15 a
Seed dormancy	0.50 ± 0.1 a	0.50 ± 0.1 a	**0.55 ± 0.05 a**	**0.63 ± 0.05 b**	0.92 ± 0.09 a	1.01 ± 0.09 a	**0.95 ± 0.24 a**	**0.77 ± 0.24 b**

*Note*: Letters and bold entries indicated statistically different (*p* < .05) results estimated by linear mixed‐effects models.

Analyzing changes in FD, we revealed a significant reduction in values for seed production in both subalpine and alpine sites. A similar pattern was detected for seed dormancy in alpine sites. Additionally, we detected a significant increase in FD for specific leaf area and leaf frost tolerance in the alpine sites. The significant shift towards trait divergence implies an increase in species with contrasting SLA and leaf frost tolerance values.

## 4 DISCUSSION

The long‐term effects of climate and land use change on mountain habitats can vary across elevational belts. Yet we have limited data on how environmental conditions and plant community and functional structure have shifted over long time periods in different elevational belts due to a lack of comprehensive and spatiotemporally replicated datasets on biodiversity in montane areas. We leverage a series of phytosociological surveys and a recent resurvey to assess how vegetation composition and functional diversity have shifted in subalpine vs. alpine elevational belts over 83 years in the Wetterstein Mountains. Subalpine and alpine sites have become increasingly thermophilus, with a greater abundance of more palatable plants and plants associated with warm and dry conditions. Although we detected remarkable stability in species occurrence over time, functional diversity has shifted in subtle ways. Specifically, species with low fecundity, high capacity for animal dispersal, and greater drought‐ and frost‐tolerance have increased in abundance. Investigating community and trait turnover in response to climate change using historical resurveys of semi‐permanent plots may be a fruitful avenue for understanding how species composition may shift with changing climate and land use regimes.

### 4.1 Signs of thermophilisation of subalpine vegetation

The Schachen area is experiencing considerable changes in macroclimate, like many uplands across the globe (e.g., Sweden's subalpine forests (Kullman, 2010), Dovrefjell, Norway (Michelsen et al., 2011)), and indeed across all 60 summits (mainly alpine and nival vegetation belts) in the European mountains (Gottfried et al., 2012). Specifically, the mean temperature of growing season is nowadays almost 2°C warmer than at the time of the first vegetation survey in the area, but precipitation has remained the same over time. As a result, higher evaporation rates could increase the frequency, duration, and severity of summer droughts amplifying water scarcity in the upland and shallow soil on porous calcareous bedrocks (Kammer & Möhl, 2018; Rosbakh et al., 2017). However, we note that this study does not assess rainfall frequency, snowfall, fog, or other moisture sources explicitly. As predicted in our first hypothesis (H1), Landolt Indicator Values (IVs) revealed thermophilisation of subalpine plots, which became drier and more palatable over time (i.e., species that preferred dry conditions and were of better forage quality increased in abundance). These environmental changes, in accordance with our second hypotheses, could explain the decline in species abundance in subalpine sites.

We did not find any support for the second part of our first hypothesis, regarding effects of eutrophication on vegetation in neither subalpine nor alpine sites. Due to its relatively close position to sources of nitrogen emissions from urban, agricultural and industrial areas and rising CO_2_ levels, the Schachen area should have experienced eutrophication to some extent through atmospheric nitrogen deposition (Rosbakh et al., 2021). Yet, the unaltered IV for soil nutrients contradicts the eutrophication hypothesis and suggests that the (sub)alpine grasslands were not affected by the high levels of N deposition in the past (Kirchner et al., 2014). Alternatively, even if nitrogen deposition increased nitrogen inputs, decreased nitrogen inputs from livestock (e.g., urine; Chirinda et al., 2019) could have resulted in no net change in soil nitrogen content. Furthermore, the extreme topographic heterogeneity of uplands combined with highly species‐specific responses to nitrogen fertilization (Körner et al., 1997) might mask (sub)alpine vegetation responses to increased nitrogen inputs. Finally, the IV for soil nutrients considers three macronutrients: nitrogen, phosphorus, and potassium. All three macronutrients must increase for the IV for soil nutrients to increase. However, the main soil nutrient limiting plant growth on calcareous soils is phosphorous rather than nitrogen (Litaor et al., 2005), so increased nitrogen levels are unlikely to benefit (sub)alpine grassland plant growth without concurrent increases in phosphorus (Guan et al., 2024).

### 4.2 Alpine plant diversity remains stable over the last eight decades

Despite the relative spatial proximity to the subalpine plots, our analyses of temporal beta diversity revealed that local ecological conditions in the alpine vegetation in the Schachen area and its characteristics have remained relatively constant over the past 80 years, in contrast with our second hypothesis (H2). Although warmer growing seasons likely reduced soil moisture content, species richness, occurrence, and abundance have not considerably changed since the first survey in the 1930s. Although considerable changes in alpine species richness and composition are more commonly reported (Lamprecht et al., 2018; Pauli et al., 2012; Steinbauer et al., 2018), other studies have detected no changes in overall species richness in alpine areas (Keller et al., 2000; Vittoz et al., 2009; Windmaiβer & Reisch, 2013). Several reasons could underlie this finding. First, many studies reporting changes in alpine species composition are based on floristic surveys in high mountain regions with low individual density (i.e., many unoccupied sites), allowing for migration of subalpine species to higher elevations (Windmaiβer & Reisch, 2013). The grasslands studied here lack unoccupied sites, limiting microsites for germination and thus potentially limiting migration (“initial floristic concept” sensu Egler, 1954). Second, although grazing density has declined, alpine sites still experience some grazing in the Schachen. Continued presence of grazers could contribute to the stability of plant occurrence and abundance in the region. Third, climate warming can open new habitats for species from low elevations without losing the coldest habitats due to topographic heterogeneity (Körner & Hiltbrunner, 2021; Scherrer & Körner, 2011). Microtopography may allow other drivers such as soil conditions, nutrient availability, and eutrophication to affect species composition. Fourth, seed banks may contribute to the consistency of alpine plant communities (Ma et al., 2010, 2020), Finally, the longevity of many alpine species could result in constancy over time since it allows continuous occupation of vegetation gaps, promotes long extinction lags, and stabilizes the existing community (Dullinger et al., 2012; Körner & Hiltbrunner, 2021; Rumpf et al., 2019; Schweingruber & Poschlod, 2006; Svenning & Sandel, 2013; Windmaiβer & Reisch, 2013; Witte et al., 2012).

### 4.3 Plant traits responses to long‐term decrease in soil moisture and changes in grazing regimes

We detected several changes in functional trait composition over time in both the alpine and subalpine belts, in concordance with our third hypothesis (H3). Here, we discuss potential links between trait responses and changes in both regional climate conditions and local environmental conditions (measured using IVs). However, we note that we are unable to link trait change directly to hypothesized environmental change in subalpine vs. alpine plots because we used IVs to estimate environmental conditions, which were estimated from the same suite of species.

Species producing fewer seeds (lower fecundity) increased in both alpine and subalpine sites. This pattern was detected using both CWMs and FD, meaning that both mean and variation in seed production decreased. Seed production is highly sensitive to environmental stressors such as water, temperature, and nutrient supply (Jump & Woodward, 2003; Rosbakh et al., 2018; Salisbury, 1942; Vaupel & Matthies, 2012; Walters & Reich, 2000) so decreases in soil moisture and/or warming temperatures in this region likely limit seed production. Similarly, upland species in the Schachen produce lower seed set under experimental extreme drought (Rosbakh et al., 2017). Our findings imply that species with high fecundity may be maladapted to current (dry) ecological conditions and are filtered out of the plant community.

Species with higher potential for dispersal, especially by epizoochory (attachment of plant propagules to animal body surfaces) increased in both alpine and subalpine sites. Epizoochory could be a vector for dispersal into alpine plots if seeds adhere to cattle, sheep, small mammals (e.g., marmots, ibex, chamois), or to domestic livestock such as cattle, horses, or sheep (Römermann et al., 2005; Rosbakh et al., 2022). The Schachen has experienced a shift in grazing system over time. Specifically, grazing shifted from cattle to sheep over the course of this study, potentially allowing greater attachment of seeds to wool. Additionally, endozoochory (carrying plant propagules in the gut) increased in alpine sites. Species such as alpine chamois (*Rupicapra rupicapra*) ingest seeds and disperse them across the alpine belt (Dullinger et al., 2013). These findings suggest that understanding changes in alpine vegetation under anthropogenic change would benefit from integrating information on multiple dispersal vectors (Poschlod & Bonn, 1998).

Species with higher frost and drought tolerance increased in alpine sites. Specifically, species with higher foliar frost tolerance and deeper seed dormancy increased in dominance. In other words, species better adapted to harsh, dry, cold environments increased. These trait shifts both corresponded with regional climate trends wherein temperatures warmed while precipitation remained constant. First, increased evaporation rates may have exacerbated summer droughts. Frost tolerance shares an ecophysiological mechanism with drought tolerance (Visakorpi et al., 2024). For example, higher concentrations of osmolytes help plants withstand low temperatures as well as maintain high osmotic pressure (Dubrovina et al., 2015; Hajihashemi et al., 2020; Ugarte et al., 2021). Second, as temperatures warm, snow melts out earlier, increasing plants' probability of experiencing frost events. Higher frost tolerance could promote survival under these freezing temperatures after snow insulation is removed. Species with deeper seed dormancy might have a higher probability of persisting in harsh alpine conditions if they can delay germination until conditions are suitable (Cohen, 1967; Gremer et al., 2016; Mondoni et al., 2012). Moreover, the increase in community weighted means for seed dormancy and concurrent decrease in functional diversity of seed dormancy suggests that environmental stress (e.g., frost events and drought) may select for species with deeper dormancy which need a longer period of cold stratification.

Finally, functional diversity in specific leaf area (SLA) and foliar frost tolerance increased in alpine sites. SLA, like frost and drought tolerance, is related to water availability (Poorter et al., 2009). Drought‐ and frost‐tolerant species typically have lower SLA values (Hamann et al., 2018); indeed, this pattern holds in the Schachen (Bucher & Rosbakh, 2020). The increase in functional diversity of SLA and foliar frost tolerance in the alpine belt may be driven by the increasing number of drought‐ and frost‐tolerant species. Increasing functional diversity (i.e., trait divergence) in SLA and foliar leaf tolerance implies an increase in species with wider variation in SLA and leaf frost tolerance. In this case, alpine communities would typically have low community‐weighted mean SLA (Scheepens et al., 2010), but variation in SLA is increasing as thinner‐leaved, faster‐growing species increase in dominance even as thick‐leaved, slow‐growing species remain in the community. Similarly, more variation in foliar frost tolerance suggests a rise in dominance for frost‐ and drought‐tolerant species. Since the alpine sites studied here have not experienced species losses, this data suggests that the vegetation within the Schachen's alpine belt is showing increasingly mixed growth and persistence strategies.

### 4.4 Importance of incorporating historical resurveys and multiple diversity metrics in understanding changes in biodiversity

Historical (re)surveys are a valuable source of information on baseline conditions pre‐intensive anthropogenic change. Moreover, historical datasets can be used to measure temporal changes in vegetation abundance and community structure that may not be reflected in measurements of species occurrence or richness. For example, we detected no temporal changes in species occurrence but some evidence for changes in species abundance in subalpine plots. We also detected significant changes in community functional trait composition and functional diversity, especially in the alpine belt. This illustrates that functional trait metrics like community weighted means and functional diversity can capture invisible changes not shown by classic biodiversity indices such as TBI. Ultimately, the variation in our results using TBI in species occurrence and abundance and community functional trait values, as well as the discrepancy between landscape‐scale loss of montane grasslands and limited species loss at a smaller scale within montane grasslands, indicate that studies not incorporating a comprehensive view of diversity should be evaluated with care. Our study demonstrates that historical records can be combined with ecological indictors to assess past conditions as well as both classic and functional diversity indices to take a holistic approach to examine long‐term vegetation changes.

## AUTHOR CONTRIBUTIONS

**Meredith A. Zettlemoyer:** Writing – original draft (lead); writing – review and editing (lead). **Svenja Munck:** Conceptualization (supporting); data curation (supporting); formal analysis (supporting); investigation (lead); methodology (supporting); project administration (supporting); writing – review and editing (supporting). **Peter Poschlod:** Conceptualization (equal); funding acquisition (supporting); methodology (supporting); project administration (supporting); resources (supporting); supervision (supporting); writing – review and editing (supporting). **Sergey Rosbakh:** Conceptualization (lead); data curation (lead); formal analysis (lead); funding acquisition (lead); investigation (equal); methodology (equal); project administration (lead); resources (lead); supervision (lead); visualization (lead); writing – review and editing (supporting).

## FUNDING INFORMATION

None.

## CONFLICT OF INTEREST STATEMENT

The authors have no conflicts of interest to declare.

## Supporting information


Appendix S1.

## Data Availability

Data used in this study will be available in the ReSurveyEurope database upon publication and in the figshare data repository at 10.6084/m9.figshare.25857415.
