# Supplementary material for: Subtle changes in plant diversity in the Bavarian Alps over the past eight decades
Source: Ecol Evol. 2024 Sep 2;14(9):e70035. doi: 10.1002/ece3.70035 (PMC11366975; doi:10.1002/ece3.70035)

**SUPPLEMENT**

**Table S1.** Transformation of the Hult-Sernander-Du Rietz scale (as modified by Niilo Söyrinki) into the Braun-Blanquet (1966) method.

| **Hurt-Sernander-Du Rietz (mod. Söyrinki)** | | **Braun-Blanquet** | |
| --- | --- | --- | --- |
| **Symbol** | **Coverage (%)** | **Symbol** | **Coverage (%)** |
| + | More than zero | r | 1 individual |
| 1- |  | + | <1 |
| 1 | <5 | 1 | <5 (6-50 individuals) |
| 1+ |  | 2m | <5 (>50 individuals) |
| 2- |  | 2m | <5 (>50 individuals) |
| 2 | <12 | 2a | <15 |
| 2+ |  | 2a | <15 |
| 3 | <25 | 2b | <25 |
| 3+ |  | 2b | <25 |
| 4 | <50 | 3 | <50 |
| 5 | <100 | 5 | <100 |

**Table S2.** Seven community types described by Niilo Söyrinki, defined by the dominant species in each type. The seven community types can be broadly grouped as subalpine vs. alpine. We provide the number of plots of each community type resurveyed in 2019 (N=43 plots).

| **Community type** | **Number of plots** |
| --- | --- |
| **Subalpine** |  |
| *Caricetum ferruginea* | 5 |
| *Ranunculus aconitifolius* | 10 |
| *Nardetum strictae* | 4 |
| *Sesleria albicans – Anthyllis vulneraria* | 3 |
| **Alpine** |  |
| *Bistorta vivipara - Silene acaulis* | 5 |
| *Caricetum firmae* | 7 |
| *Salicetum herbaceae* | 9 |

**Table S3.** Changes in environmental conditions during the growing season (April – September, the months during which the majority of plants frow, flower, fruit, and senesce) over time in the Schachen region of the Wetterstein Mountains, Bavarian Alps, Germany. We included either (A) air temperature (°C) or (B) precipitation (mm) as response variables and year and year^2^ as predictors in two separate ordinary least squares regressions (*nlme* R package). Air temperature (2m above the ground) and precipitation data were obtained from the closest weather station ‘Zugspitze’ located 11km west of the Schachen area at 2962m a.s.l. (www.dwd.de). ***p<0.0001.

|  | 1. **Air Temperature** | | 1. **Precipitation** | |
| --- | --- | --- | --- | --- |
|  | Coefficient ± SE | t-value | Coefficient ± SE | t-value |
| Year | -2.737 ± 0.650 | -4.209 *** | 2.7048 ± 46.80 | 0.0578 |
| Year^2^ | 0.0007 ± 0.0002 | 4.234 *** | -0.0006 ± 0.01 | -0.049 |
| Residual (df) | 0.0062 (84) |  | 0.0046 (84) |  |

**Table S4.** Changes in environmental Indicator Values (IVs) in (A) subalpine and (B) alpine plots over time. We conducted separate linear mixed models for each EIV: (1) Landolt’s T (a proxy for mean soil and surface temperature after snowmelt), (2) Landolt’s F (a proxy for soil moisture), (3) Landolt’s N (a proxy for soil fertility), and (4) Briemle et al. 2002’s forage quality value (FQ) (where 1=low forage quality and 9=high forage quality). Each model included a community weighted mean IV as the response variable, survey year (1936 vs. 2019) as a predictor, and community type (Table S2) as a random factor. Analyses were conducted separately for subalpine vs. alpine sites because of the relatively small number of replicate plots. ***p<0.0001; **p<0.01; *p<0.05; •p<0.1.

|  | 1. **Subalpine** | | 1. **Alpine** | |
| --- | --- | --- | --- | --- |
|  | **Coefficient** ± **SE** | **χ^2^** | **Coefficient ± SE** | **χ^2^** |
| **(1) Landolt’s T** |  |  |  |  |
| Survey year (2019) | 0.0728 ± 0.0299 | 5.94 * | 0.0912 ± 0.0580 | 2.47 • |
| Residual | 0.0098 |  | 0.0353 |  |
| Community type | 0.0082 |  | 0.0306 |  |
| **(2) Landolt’s F** |  |  |  |  |
| Survey year (2019) | -0.0848 ± 0.0288 | 8.69 ** | -0.0709 ± 0.0254 | 7.81 ** |
| Residual | 0.0091 |  | 0.0068 |  |
| Community type | 0.1542 |  | 0.0183 |  |
| **(3) Landolt’s N** |  |  |  |  |
| Survey year (2019) | -0.0301 ± 0.0435 | 0.48 | -0.1713 ± 0.0971 | 3.11 • |
| Residual | 0.0208 |  | 0.0990 |  |
| Community type | 0.4761 |  | 0.1605 |  |
| **(4) FQ** |  |  |  |  |
| Survey year (2019) | 0.3037 ± 0.0704 | 18.63 *** | -0.0216 ± 0.0893 | 0.06 |
| Residual | 0.0545 |  | 0.0837 |  |
| Community type | 0.1452 |  | 0.1655 |  |

**Figure S1.** Study area in the Wetterstein Mountains, Bavarian Alps, Germany (ArcGIS Desktop v.10.4.1; ESRI). Black symbols indicate alpine plots; gray symbols indicate alpine plots. Shapes represent different communities within the alpine and subalpine belts (see Table S2).


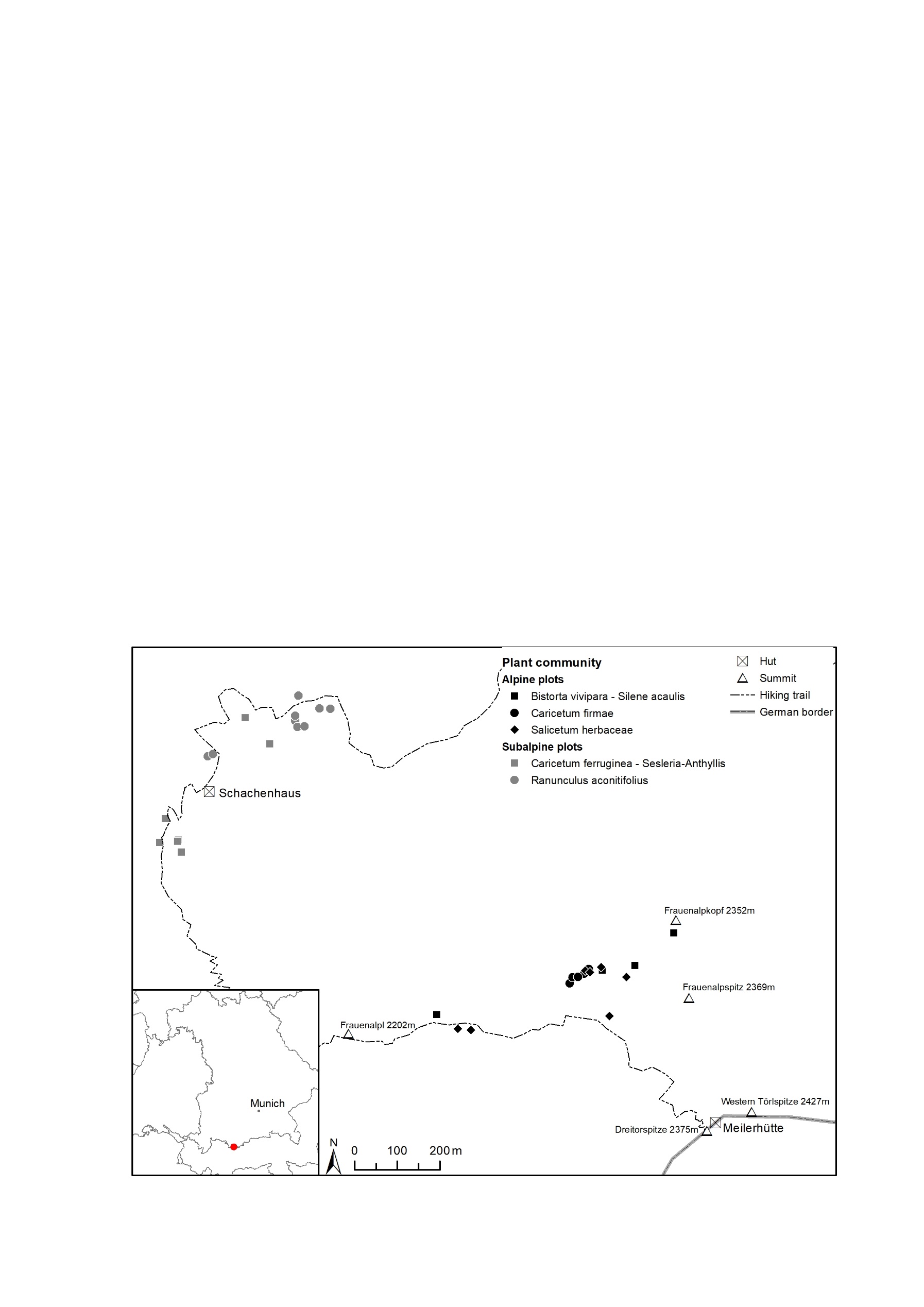

Supplement: Supplementary file 1 — Appendix S1. [file ECE3-14-e70035-s001.docx]
